# The Impact of Polygenic Risk, Parental Separation, and Parental Relationship Discord on Heavy Episodic Drinking Across Adolescence and Young Adulthood in a High-Risk Sample

**DOI:** 10.1016/j.jaacop.2025.06.001

**Published:** 2025-06-09

**Authors:** Sally I-Chun Kuo, Vivia V. McCutcheon, Kathleen K. Bucholz, Danielle M. Dick, Fazil Aliev, Jacquelyn L. Meyers, Sarah J. Brislin, Grace Chan, Howard J. Edenberg, Chella Kamarajan, John Kramer, Samuel Kuperman, Dongbing Lai, Martin H. Plawecki, Carolyn E. Sartor, Marc A. Schuckit, Jessica E. Salvatore

**Affiliations:** a Robert Wood Johnson Medical School, Rutgers University, Piscataway, New Jersey; b Washington University School of Medicine, St. Louis, Missouri; c SUNY Downstate Health Sciences University, Brooklyn, New York; d University of Connecticut School of Medicine, Farmington, Connecticut; e Indiana University School of Medicine, Indianapolis, Indiana; f University of Iowa Carver College of Medicine, Iowa City, Iowa; g University of California San Diego School of Medicine, La Jolla, California

**Keywords:** parental divorce, parental conflict, heavy episodic drinking, trajectories, polygenic scores

## Abstract

**Objective:**

Parental separation and relationship discord are linked to alcohol use behaviors, but their influence on the longitudinal course of alcohol misuse and interactions with genetic predisposition remain unclear. This study examined how the longitudinal course of heavy episodic drinking (HED) from adolescence to young adulthood varies with polygenic risk, parental separation, and relationship discord.

**Method:**

Participants were from the Collaborative Study on the Genetics of Alcoholism (COGA) Prospective Sample, and included individuals from 2 genetically inferred continental groups: European-like (EA; n = 1761) and African-like (AA; n = 894) who were reassessed biennially (mean age = 16.39 at first assessment; mean assessments = 4.65). Alcohol misuse was indexed by past-year HED frequency. Predictors included parental separation, parental relationship discord, and problematic alcohol use polygenic scores (PGS_PAU_). Data were analyzed using linear mixed-effects growth models.

**Results:**

HED increased through young adulthood before declining. In European Americans (EA), parental separation was associated with HED intercepts, but not with linear slope or quadratic curvature. Higher PGS_PAU_ was associated with a faster initial growth and slower decline. In African American (AA), parental relationship discord was not associated with HED intercepts but was associated with a faster initial growth and slower decline. PGS_PAU_ were not associated the intercept or the course of HED. No interaction was found between PGS_PAU_ and parental separation or discord to predict the longitudinal course of HED in either EA or AA samples.

**Conclusion:**

Genetic risk and exposure to parental separation and discord are associated with the course of HED, with some differences across continental groups.

## Plain language summary

This study utilized data from the Collaborative Study on the Genetics of Alcoholism (COGA) to examine how the course of heavy episodic drinking (HED) from adolescence to young adulthood varies with genetic risk, parental separation, and relationship discord. Parental separation and relationship discord were associated with initial levels of heavy episodic drinking and their course into young adulthood. In the European American sample, these family stressors were associated with higher initial levels of heavy episodic drinking, which were sustained over time with genetic factors amplifying this pattern. In the African American sample, parental relationship discord was associated with a rapid increase in heavy episodic drinking that declined slowly. These findings highlight the contributions of genetics and family adversity in shaping risk for harmful patterns of alcohol use across development.

Parental divorce and marital discord are stressors frequently encountered by children and adolescents, and both are linked to an elevated risk for alcohol misuse and related problems.^1^, ^2^, ^3^ Annually, around 1 million children and adolescents experience parental marital dissolution,^4^ and by age 18 years, approximately 28% of Americans experience parental separation/divorce.^5^ Even without parental separation, many children are exposed to parental marital conflicts.^6^ Extensive research indicates that parental divorce and discord correlate with an increased risk for a range of poorer adjustment among offspring.^7^^,^^8^ Given their prevalence and significant economic, psychological, and social costs^9^, understanding their impact on children’s development remains a priority for families, educators, clinicians, researchers, and policymakers.

It is well established that parental divorce and parental discord are linked to indicators of alcohol misuse in offspring. These indicators include early age at initiation,^1^ increased levels of alcohol misuse that start in adolescence and persist into adulthood,^10^ and the development of alcohol use disorder (AUD).^11^^,^^12^ However, a limitation in these studies is the predominance of cross-sectional data, which typically rely on adult participants’ retrospective reports of alcohol use during adolescence. This approach is limited because alcohol use/misuse is a developmental phenomenon exhibiting age-related patterns; misuse typically begins in adolescence, peaks in the early 20s, and declines thereafter.^13^ Cross-sectional studies may miss the dynamic pattern of alcohol use. Moreover, AUD signifies a clinical endpoint in a progression influenced by various factors evolving over time.

There is also a related need to understand how parental separation and discord interact with genetic factors to influence the course of alcohol misuse behaviors over time. Genetic factors play an important role in alcohol use behaviors.^14^ In addition, there is evidence of shared genetic influences on both AUD and divorce.^15^ This suggests that youth exposed to parental divorce/separation and discord may have higher genetic risk for alcohol problems, in addition to more exposure to environmental risks such as parental alcohol misuse and less stable home environments. Furthermore, growing evidence suggests that genetic influences on alcohol outcomes varies as a function of environmental conditions, including stress, family environments, and parenting.^16^ Consistent with the idea that socio-environmental contexts can trigger a genetic diathesis,^17^ in a recent report we found stronger genetic effects on men’s alcohol consumption trajectories among those who experienced parental divorce compared to those who did not.^18^ This suggests that parental separation and discord may alter the effect of genetic predispositions on alcohol misuse over time. Understanding how genetic factors and family stressors such as parental separation and discord shape the longitudinal course of alcohol misuse may further our understanding of why some individuals may be at particular risk for poorer outcomes following these family stressors.

In this study, we adopted a developmental perspective and tested whether there are gene-by-environment interactions whereby parental separation, parental relationship discord, and polygenic predispositions for problematic alcohol use jointly influence the longitudinal course of heavy episodic drinking (HED) from adolescence to young adulthood. We tested this in a high-risk longitudinal sample from the Collaborative Study on the Genetics of Alcoholism (COGA). To index genetic risk, we used a polygenic scoring approach, recognizing the polygenic architecture of alcohol outcomes.^19^ In addition, we conducted stratified analyses for European Americans (EA) and African Americans (AA), broad continental groups statistically inferred from genomic data. These groupings broadly correspond with self-reported race and act as a proxy stratifying variable^20^ for sociocultural factors^21^ that may influence variation in the course of alcohol use behaviors^22^ or the associations between environmental adversities and alcohol use outcomes.^23^, ^24^, ^25^ We tested 2 preregistered hypotheses (osf.io/gr2qm):

1. Higher problematic alcohol use polygenic scores, parental separation, and parental relationship discord will be associated with a higher baseline level of HED and a more rapid escalation of HED over time.
2. Parental separation and parental relationship discord will each moderate the association of problematic alcohol use polygenic scores and the longitudinal course of HED such that exposure to parental separation and parental relationship discord will accelerate HED among individuals with higher genetic predispositions for problematic alcohol use.

## Method

Participants came from the Collaborative Study on the Genetics of Alcoholism (COGA) prospective study.^26^ COGA is a diverse family-based study the objective of which is to identify genetic and environmental factors implicated in the development of AUD and related psychiatric disorders. Probands were identified through alcohol treatment centers across 6 sites in the United States, with a seventh site added for one wave. Probands along with their families were invited to participate if the family was sufficiently large (usually sibships greater than 3 with parents available), with 2 or more members living in the COGA site’s catchment area. Comparison families not selected for the presence or absence of alcohol problems were recruited from the same communities. Beginning in 2004, a sample of adolescent and young adult offspring (12-22 years of age) of prior adult COGA participants from the initial 6 sites were recruited into the COGA prospective study on the development of alcohol use disorders and related problems.^26^ Participants were interviewed at enrollment and followed up at approximately biennial intervals. The Institutional Review Board at all data collection sites approved the study, and written consent (and assent for adolescents whose parents had provided consent) was obtained from all participants.

The present study made use of data from European Americans (EA) and African Americans (AA), broad continental groups statistically inferred from genomic data and the 2 largest continental populations represented in COGA. Continental group assignments were statistically inferred by estimating how similar an individual’s genetic data were to the genomes of 1000 Genomes^27^ populations from the following: (1) Northern Europeans living in Utah, individuals from Finland, Great Britain, Toscani from Italy, and Iberians from Spain, contributing to a European-like group; and (2) African-Caribbeans in Barbados, Gambians from the Western Division, Esan in Nigeria, Mende in Sierra Leone, Yoruba in Ibadan, Luhya in Webuye, and African ancestry individuals in southwestern United States contributing to an African-like group. Final continental assignment was ascribed on a per family basis as the assignment for most individuals within the family.^28^ The analytic sample included 1761 EA individuals (51% male; mean age = 16.41 years, SD = 3.29 at first assessment) and 894 AA individuals (52% female; mean age = 16.36 years, SD = 3.31 at first assessment). We note that the concordance between continental group and self-report race–ethnicity is high (>97%) in COGA. Participants had, on average, 4.71 assessments (SD = 1.99) and 4.61 assessments (SD = 1.81) in the EA and AA samples, respectively.

### Measures

#### Frequency of Heavy Episodic Drinking

Frequency of heavy episodic drinking (HED) was coded from participants’ response to the question, “How often did you have five or more drinks in 24 hours during the last 12 months?” from the Semi-Structured Assessment for the Genetics of Alcoholism (SSAGA) Interview.^29^ Responses included 13 options, ranging from “never,” “about 2 days a week” (100-149 days), to “every day.” Response options were converted into frequencies by taking the midpoint of each response option.^30^ For example, the responses “2 days per week” (100-149 days) and “every day” were translated to 124.5 days and 365 days per year, respectively. Those who reported that they did not drink during the past year were coded as zero.

#### Parental Separation

Parental separation was based on participant report of not living with both biological parents for the majority of time from between ages 12 to 17 years^31^ from the Home Environment section of the SSAGA.^29^ The construct was coded as parental separation = 1 for those who reported that they did not live with both biological parents most of the time between ages 12 and 17 years, and 0 for all others.^32^ We note that although the SSAGA has additional questions regarding whether mother/father were absent because of marital separation/divorce/desertion, these questions were not systematically asked of all participants because of a skip pattern in the interview. Thus, we opted to use the question regarding living arrangements while growing up (ie, not living with both biological parents) as our indicator of parental separation/divorce, which all participants were asked.

#### Perceptions of Parental Relationship Discord

Participants reported on their perceptions of parental relationship discord using 5 questions. These items asked participants about the quality of their parents’ marriage/relationship (rated on a 4-point scale, from “poor” to “excellent”) and how much conflict or tension there was in the household (rated on a 4-point scale, from “none” to “a lot”); and 3 yes/no questions: whether their parents usually seemed to enjoy each other; whether their parents often argued or fought in front of them; and whether either of their parents ever hit the other. Participants who reported living in a single-parent household without shared custody were not asked questions about their perceptions of parental relationship discord. Based on prior work showing items loaded onto a single factor,^24^ a composite measure was calculated by taking the prorated sum of the items for participants who responded to at least 3 of these items (Supplement 1, available online). Participants over 18 years of age were asked to retrospectively report on parental relationship discord between ages 12 and 17 years. We used participants’ reports from their baseline assessment for analyses, with the majority (60.2%) providing concurrent baseline reports.

#### Problematic Alcohol Use Polygenic Scores

A full description of genotyping, data processing, quality control, and imputation is provided in Lai *et al.*^28^ (Supplement 1, available online). To avoid confounding due to population stratification,^33^ we conducted analyses separately for the EA and AA groups. Within-group continuous genetic similarity principal components were estimated from GWAS data using Eigenstrat^34^ and the 1000 Genomes, Phase III reference panel.

Genetic risk for problematic alcohol use was indexed by constructing genome-wide polygenic scores (PGS), which are aggregate measures of the number of risk alleles that individuals carry weighted by effect sizes from GWAS summary statistics. We used PRS-CSx^35^ to construct the polygenic scores. PRS-CSx^35^ uses ancestry-specific discovery sample GWAS weights, paired with linkage disequilibrium information from an ancestry-matched external reference panel, to estimate the posterior effect size for each SNP. For EA participants, we used discovery sample GWAS summary statistics for problematic alcohol use in individuals genetically similar to reference populations from Europe (ie, EA) from a recent analysis of GWAS meta-analysis of problematic alcohol use^36^ (COGA removed). For AA participants, we used GWAS summary statistics from a GWAS meta-analysis summary statistics in EA^36^ in tandem with GWAS summary statistics from recent GWAS meta-analyses of problematic alcohol use in individuals genetically similar to reference populations from Africa (ie, AA)^36^ (COGA sample removed). Because PRS-CSx improves predictive power for non-European ancestry samples with smaller GWAS,^35^ we included both the European and African ancestry–derived polygenic scores in the AA sample in COGA, but only the European ancestry–derived polygenic scores in the EA sample in COGA. To account for population stratification, we regressed polygenic scores on the first 10 genetic similarity principal components (PC1-PC10) within each group (EA and AA) and used the residualized standardized polygenic scores in analyses.

### Analytic Plan

We ran a series of multilevel linear mixed models (MLM) to examine the associations between problematic alcohol use polygenic scores (PGS_PAU_), parental separation, parental relationship discord, and the longitudinal course of HED. Our decision to use a multilevel approach over other longitudinal approaches such as latent growth curves was guided by the fact that an MLM can accommodate unequal spacing between time intervals and unbalanced data (ie, it does not require each individual to be observed at every chronological age).^37^

We used age as the metric of time, and polynomial age terms (ie, linear, quadratic) were included to describe the linear and quadratic change in heavy episodic drinking over time. The categories at the tail ends of the age spectrum were collapsed because of small cell sizes, such that they ranged from 12 (and below) to 32 (and above). Heavy episodic drinking was log transformed to adjust for skewness. Mixed effects models were conducted using the *lme4* package^38^ for R.^39^ Because COGA included related individuals, we fit 2-level models (observations nested within individuals over time) and robust standard errors to adjust for familial clustering.

We conducted analyses in 3 stages. We first determined the longitudinal course of heavy episodic drinking over the course of time, comparing the goodness of fit to linear and polynomial models (quadratic). We used model fit statistics, including the Akaike information criterion (AIC) and likelihood ratio tests to assess improvement in fit. Second, we examined the appropriate structure of the random-effects components of the model using nested models. We estimated random effects using restricted maximum likelihood (REML). We used AIC and likelihood ratio tests to assess model fit improvement. Finally, we examined our main research questions (focal analyses)—that is, do PGS_PAU_, parental separation and parental relationship discord influence the longitudinal course of heavy episodic drinking from adolescence to young adulthood? Do parental separation and parental relationship discord modify the influence of PGS_PAU_ on the longitudinal course of heavy episodic drinking over time? Prior to running the focal analyses, all continuous predictors were centered at their means, which improves the interpretability of the intercept. Furthermore, we examined the interactive effects between PGS_PAU_ and parental separation and parental relationship discord by including product term between mean-centered 2-way interaction terms in the model. Models included sex and parental education (Supplement 1, available online) as covariates.

To account for differences in patterns of drinking and broad sociocultural factors,^21^ focal analyses were stratified by genetically inferred continental groups (ie, EA and AA). These groupings broadly aligned with self-identified race and serve as a proxy stratifying variable.^20^ Separate models were fit for parental separation and parental relationship discord in view of prior evidence that parental separation and quality of the parental relationship exert separate and unique effects on children’s adjustment.^40^ For all *a priori* hypotheses, we used a *p-*value threshold of *p* < .05 for inference criteria.

## Results

### Descriptive Statistics

Table 1 summarizes descriptive statistics for the key study variables, separately for EA and AA. In the EA sample, age at initiation of regular drinking was 17.08 years. Approximately 40% of the EA sample was exposed to parental separation. In the AA sample, age at initiation of regular drinking was 18.07 years. Approximately 66% of the AA sample experienced parental separation. Table S1, available online, presents mean and standard deviation for frequency of HED by age and ancestry. Table S2, available online, provides zero-correlations for EA and AA separately. Although parental discord and parental separation were related, they represent distinct constructs. Parental discord was only moderately correlated with parental separation in both the EA (*r* = 0.31, *p* < .05) and the AA (*r* = 0.23, *p* < .05) samples.

**Table 1:** Descriptive Statistics for Key Study Variables in the European American and African American Participants

	EA	AA
	Mean (SD) or %	Mean (SD) or %
Participants, n	1,761	894
Sex, female	51%	52%
Age at first assessment	16.41 (3.29)	16.36 (3.31)
Age at last assessment	24.80 (4.87)	24.99 (4.56)
No. of assessments	4.71 (1.99)	4.61 (1.81)
Age at initiation of regular drinking	17.08 (2.78)	18.07 (2.88)
Parental education, y	13.91 (2.14)	12.29 (1.84)
Parental separation	40%	66%
Parental discord	1.00 (1.15)	1.11 (1.12)
Parental history of alcohol use disorder	75%	69%
Total number of observations, N	8,293	4,125

Note: Mean and SD are provided for continuous measure, and percentage is provided for dichotomous measure. Parental education was measured using the highest level of educational attainment (years) of either parent.

### Linear Mixed Models of the Longitudinal Course of Heavy Episodic Drinking

**Figure 1:**
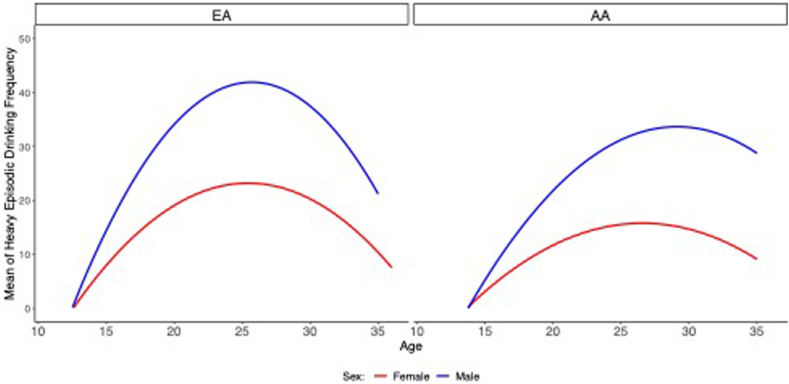
Mean Frequency of Heavy Episodic Drinking Across Age for Female Participants (Red) and Male Participants (Blue) Quadratic Fitted Function, Separately for European American (EA) and African American (AA) Groups

Figure 1 presents the mean for frequency of HED across the age range by sex, separately for EA and AA groups. Results showed quadratic growth across development, with sex differences. Overall, male participants showed higher levels of HED across time, and more rapid escalation. Both male and female participants showed the normative reduction in HED in the late 20s. Table 2 provides the comparisons of model fit for various multilevel growth models for HED. Results from model fitting supported the observations from visual inspection, with the inclusion of linear and quadratic terms improving overall model fit. In addition, we estimated random effects using restricted maximum likelihood (REML). Each model improved on the fit from the previous. Thus, for all subsequent focal analyses, we fit all models with a random intercept and random slopes for age and age^2^.

**Table 2:** Models for Determining Trajectories Shape and Random Effects Structure

Fixed effects model fitting	AIC	LL	df	Constant	Age	Age^2^
Unconditional model	46,547.2	–23,270.6	0	**1.408**	—	—
Linear growth model	45,318.1	–22,655.1	1	**0.486**	**0.106**	—
Quadratic growth model	44,281.9	–22,135.9	2	**–0.431**	**0.366**	**–0.013**
**Random effects model fitting**	**AIC**	**LL**	**Δdf**	**Δ** χ**^2^**	***p***	
Random intercept	44,282	–22,136	—	—	—	
Random slope for age	43,533	–21,759	2	752.97	<.001	
Random slope for age^2^	42,863	–21,422	3	675.83	<.001	

Note: Boldface type indicates estimate *p* < .05. AIC = Akaike information criterion; LL = log likelihood.

### Analyses Predicting Intercept, Slope, and Quadratic Curvature of the Longitudinal Course of Heavy Episodic Drinking

Table 3, Table 4 present the results from the main effects multilevel growth models of HED as a function of PGS_PAU_ and parental separation and discord for the EA and AA samples, respectively. All results indicated that there was linear and quadratic change in HED across time. Age was associated with these trajectories, with a positive slope, followed by a small negative quadratic effect. Being male was associated with a steeper increase over time with a small negative quadratic effect.

**Table 3:** Linear Mixed Models Predicting Frequency of Heavy Episodic Drinking Across Time as a Function of Problematic Alcohol Use Polygenic Scores, Parental Separation, and Parental Discord in the European American (EA) Sample

	Parental separation model		Parental discord model	
	n = 1,751 (observations = 8,262)		n = 1,362 (observations = 6,600)	
Parameter	b	95% CI	b	95% CI
Age				
I	**–0.563**	**[–0.719, –0.406]**	**–0.464**	**[–0.603, –0.325]**
S	**0.383**	**[0.341, 0.426]**	**0.371**	**[0.331, 0.412]**
Q	**–0.015**	**[–0.017, –0.013]**	**–0.015**	**[–0.017, –0.013]**
Sex, male				
I	**–0.232**	**[–0.432, –0.033]**	**–0.189**	**[–0.383, –0.006]**
S	**0.182**	**[0.128, 0.236]**	**0.176**	**[0.118, 0.234]**
Q	**–0.007**	**[–0.009, –0.004]**	**–0.006**	**[–0.009, –0.004]**
PGS_PAU_				
I	0.045	[–0.063, 0.152]	0.007	[–0.093, 0.124]
S	**0.035**	**[0.006, 0.064]**	**0.040**	**[0.008, 0.072]**
Q	**–0.002**	**[–0.004, –0.001]**	**–0.002**	**[–0.004, –0.001]**
Separation/discord				
I	**0.308**	**[0.097, 0.518]**	**0.128**	**[0.032, 0.224]**
S	–0.044	[–0.099, 0.012]	0.008	[–0.019, 0.035]
Q	0.001	[–0.002, 0.003]	–0.000	[–0.002, 0.001]

Note: Polygenic score residualized on the first 10 genetic similarity principal components. Separate models were run for parental separation and parental relationship discord. All models included parental education as a covariate for initial status, whereas sex was included for both initial status and change over time. Boldface type indicates estimate *p* < .05. EA = European American; I = intercept; PGS_PAU_ = problematic alcohol use polygenic score; Q = quadratic slope; S = linear slope.

**Table 4:** Linear Mixed Models Predicting Frequency of Heavy Episodic Drinking Across Time as a Function of Problematic Alcohol Use Polygenic Scores, Parental Separation, and Parental Discord in the African-Like (AA) Sample

	Parental separation model		Parental discord model	
	n = 887 (observations = 4,098)		n = 463 (observations = 2,199)	
Parameter	b	95% CI	b	95% CI
Age				
I	**–0.345**	**[–0.569, –0.121]**	**–0.285**	**[–0.461, –0.110]**
S	**0.194**	**[0.126, 0.264]**	**0.191**	**[0.147, 0.234]**
Q	**–0.007**	**[–0.010, –0.003]**	**–0.006**	**[–0.008, –0.004]**
Sex, male				
I	–0.151	[–0.384, 0.082]	–0.154	[–0.399, 0.091]
S	**0.095**	**[0.026, 0.165]**	**0.085**	**[0.022, 0.149]**
Q	–0.002	[–0.006, 0.001]	–0.001	[–0.004, 0.003]
PGS_PAU_				
I	0.012	[–0.108, 0.131]	0.012	[–0.120, 0.143]
S	–0.000	[–0.037, 0.036]	0.007	[–0.027, 0.042]
Q	0.000	[–0.002, 0.002]	0.000	[–0.001, 0.002]
Separation/discord				
I	–0.021	[–0.261, 0.218]	–0.068	[–0.180, 0.043]
S	0.013	[–0.060, 0.086]	**0.062**	**[0.034, 0.090]**
Q	0.000	[–0.004, 0.004]	**–0.003**	**[–0.004, –0.001]**

Note: Polygenic score residualized on the first 10 genetic similarity principal components. Separate models were run for parental separation and parental relationship discord. All models included parental education as a covariate for initial status, whereas sex was included for both initial status and change over time. Boldface type indicates estimate *p* < .05. I = intercept; PGS_PAU_ = problematic alcohol use polygenic score; Q = quadratic slope; S = linear slope.

#### Parental Separation

As shown in Table 3, in the EA sample, parental separation was associated with a higher HED intercept. PGS_PAU_ was associated with a more rapid escalation of HED and a less pronounced quadratic decline, indicating a slower decline in HED in the mid and late 20s. Figure S1, available online, depicts the predicted value for HED across ages 12 to 32 years by sex, conditioned on parental separation. In the interactive effects model, there was no evidence that parental separation modified the effect of PGS_PAU_ on the longitudinal course of HED. In the AA sample, as shown in Table 4, there were no significant main effects or interactive effects for PGS_PAU_ and parental separation on the intercept, linear slope, or quadratic term of HED across time.

#### Parental Relationship Discord

As shown in Table 3, in the EA sample, parental relationship discord was not associated with the intercept. In the interactive effects model, there was no evidence that PGS_PAU_ and parental relationship discord interacted to predict intercept, linear slope, or quadratic terms for HED across time. In contrast, in the AA sample, as shown in Table 4, parental relationship discord was associated with a more rapid escalation of HED and lower quadratic growth of HED (ie, slower decline in HED in the mid and late 20s). Figure S2, available online, depicts the predicted value for HED across ages 12 to 32 years by sex, conditioned on low (−1 SD) and high (+ 1 SD) parental relationship discord.

### Sensitivity Analyses

To check the robustness of the findings, we conducted a series of post hoc analyses using parental history of AUD as another method of capturing genetic risk, recognizing that parental history reflects both genetic and environmental components. Parental history of AUD was based on parent self-report when available. For those parents who were not interviewed, parental history of AUD was determined using family history reports as described in McCutcheon *et al.*^32^ We examined whether parental history of AUD and parental separation and discord were associated with the longitudinal course of HED, separately in the EA and AA samples (Tables S3, S4, available online).

In the EA sample, the patterns of associations from these sensitivity analyses were largely consistent with results from analyses using PGS_PAU_. Parental history of AUD was associated with a more rapid escalation of HED and lower quadratic growth of HED. Both parental separation and parental relationship discord were associated with higher intercepts. In the AA sample, the patterns of associations from these sensitivity analyses were virtually identical with results from analyses using PGS_PAU_. Parental history of AUD was not associated with the intercept and the longitudinal course of HED. However, in the model that included parental history instead of PGS_PAU_, parental relationship discord was associated with more rapid escalation of HED and lower quadratic growth.

## Discussion

This study expanded upon existing literature by examining how parental separation, discord, and polygenic predispositions for problematic alcohol use are associated with the course of heavy episodic drinking across adolescence and young adulthood in a high-risk, longitudinal sample of individuals from 2 genetically inferred continental groups. Consistent with prior research,^13^ we found support for a nonlinear longitudinal course of HED from adolescence through young adulthood, best described by a quadratic growth model, with an increase in HED through adolescence and the mid 20s, followed by a decline. Our findings provided a nuanced understanding of how parental separation and parental discord are associated with baseline levels and longitudinal course of HED.

In the EA sample, parental separation and parental relationship discord were associated with baseline levels of HED (intercept), but not with its escalation (linear slope) or reduction (quadratic term). This is consistent with prior evidence linking parental separation and discord to earlier alcohol milestones, such as early initiation^1^^,^^41^ and higher misuse in adolescence.^10^ Because most young adults no longer live with one or both parents, the ongoing influence of parental separation and relationship discord on daily functioning may be diminished, which could explain why these factors were associated with baseline levels of HED in adolescence but not with its rate of escalation over time across young adulthood. This underscores the impact of familial stressors on the early stages of alcohol misuse. Our results suggest that the previously noted cross-sectional variation in alcohol misuse associated with parental separation^42^ are driven largely by initial HED levels in adolescence, with these differences persisting over time.

Problematic alcohol use polygenic scores were associated with escalation (linear slope) and quadratic curvature of HED, but not baseline levels (intercept). This is consistent with prior research linking parental AUD, which has a genetic component, to accelerated adolescent heavy drinking.^43^ It also reflects broader findings that genetic factors have little influence on alcohol initiation and misuse in early adolescence but become increasingly important over time in shaping alcohol use patterns.^44^ Contrary to our expectation, we did not find evidence that either parental separation or discord modified the effect of problematic alcohol use polygenic scores on the longitudinal course of HED. This indicates that the effect of parental separation or discord on HED baseline and the effect of polygenic loading for problematic alcohol use on the linear slope and quadratic curvature were unique and independent. These findings suggest that the longitudinal course of HED over time is influenced by both children’s polygenic predispositions and their exposure to familial stressors. Although the absence of significant gene–environment interaction effects on the longitudinal course of HED was contrary to our hypothesis, our observed pattern of effects suggests that familial stressors and genetic factors jointly influence the development of alcohol use patterns through unique influences on baseline levels and how they are shaped over time.

In the AA sample, parental relationship discord was associated with a more rapid escalation of HED and slower decline across adolescence and young adulthood. In contrast, parental separation was not associated with the longitudinal course of HED. These findings remained consistent when using parental history of AUD to index genetic predispositions instead of PGS_PAU_. This pattern of findings suggests distinctive patterns, with parental relationship discord demonstrating a more pronounced impact on the course of HED in the AA sample. Although the mechanisms underlying this pattern are not well established in prior literature, it is consistent with research indicating that family disruption does not uniformly influence children’s behavioral outcomes. Previous studies have shown that the effects of parental separation/divorce on children’s outcomes tend to be more pronounced among children from families in which separation is less prevalent and thus less anticipated.^45^ In our sample, rates of parental separation were higher in the AA (66%) compared to EA (40%) samples, consistent with prior research showing higher rates of marital instability among African American families compared to European American families.^46^ Our results may partly reflect differing perceptions of parental separation among African American youth. In addition, research shows that residing in single-parent households is associated with an elevated risk for alcohol initiation in White girls but not in Black girls.^47^ These findings highlight the need for a comprehensive understanding of how familial stressors shape the longitudinal course of alcohol misuse in diverse populations, particularly in view of ethnic and racial differences in children’s responses to family stressors.^48^

In the AA sample, we did not find evidence that polygenic loading for problematic alcohol use was associated with the longitudinal course of HED or modified the effects of familial stressors on the longitudinal course of HED. These null effects likely reflect reduced statistical power due to smaller sample size and the limited predictive power of polygenic scores in African-like populations, stemming from the historic exclusion of diverse populations in genome-wide association studies.^49^ This highlights the need for well-powered genetic risk scores in diverse populations to advance research on genetic and familial influences on alcohol misuse trajectories and to ensure that gains from research are shared equitably across all groups. To address the issue of predictive power of polygenic scores in the AA sample, we conducted post hoc sensitivity analyses by using parental history of AUD to index genetic risk. Contrary to our expectation, parental history of AUD was also not associated with the longitudinal course of HED at a statistically significant level. We note that in the context of our high-risk sample, the majority of the AA sample had a parental history of AUD. Accordingly, limited variation in this measure may have also limited statistical power for our inferential analyses.

Our results should be considered in light of several limitations. First, COGA is a high-risk sample, with most participants from extended families enriched for AUD. In addition, parental separation was more prevalent in our sample (relative to 28% in the general population^5^), which may limit generalizability to other populations with different risk profiles. Second, our measure of parental relationship discord was based on offspring reports and may not fully reflect the actual quality of marital relationship between parents, but it does indicate how adolescents perceive it. This may be what matters for capturing risk, although it is not clear whether perceived or actual parental discord drives the association. Incorporating information from additional reporters or methods could address this possibility. Third, the timing of parental separation was not assessed, although it is important given that younger children typically face more challenges following parental separation.^50^ Fourth, because young adult participants were asked to retrospectively report on parental relationship discord, whereas adolescent participants provided concurrent reports, it was not feasible to treat parental relationship discord as a time-varying predictor. Future research that takes into account the length and severity of the exposure to parental relationship discord may be important. Fifth, parental separation was defined based on offspring reports of not living with both biological parents for the majority of adolescence. This definition reflects living arrangement rather than parental legal marital status, which is an important distinction when interpreting the findings. Furthermore, we acknowledge that this variable may reflect a range of living arrangements beyond parental separation, including parental loss, adoption, or other circumstances. Future research should consider more detailed measures to differentiate between these experiences and their potential implications. Sixth, parental discord and separation may serve as proxies for broader familial stressors, such as family conflict, parenting, or household chaos, which were not directly assessed in the present study. Future research should examine whether the observed associations persist after accounting for these additional factors related to familial dysfunction. Seventh, despite the longitudinal design of the study, not all participants contributed data spanning the entire age range under investigation. Although we used a multilevel growth modeling approach to handle unstructured time data, we recognize that this method may not fully capture the substantial heterogeneity in individuals’ longitudinal course of HED across development. Finally, we conducted analyses separately by genetically inferred continental group (EA and AA). We recognize that both broad continental group and self-identified race are imprecise proxies for the complex sociocultural and environmental factors that may contribute to differences across groups. Thus, our findings should be interpreted with caution. Although this study does not explore the underlying sociocultural factors, such as experiences of discrimination or systemic stressors, investigating these factors in future research will be important for advancing health equity.

In conclusion, in a high-risk sample enriched for familial risk of alcohol use disorder, we found that parental separation and parental relationship discord were associated with the initial level and longitudinal course of heavy episodic drinking across adolescence through young adulthood, with some variation by continental group. In the EA sample, both parental separation and discord were associated with higher initial levels for heavy episodic drinking, which persisted over time. In the AA sample, parental relationship discord was associated with faster escalation and slower decline in HED. In addition, polygenic predispositions for problematic alcohol use were associated with faster growth in HED. These results highlight the impact of familial stressors on the risk of alcohol misuse across development.

## CRediT authorship contribution statement

**Sally I-Chun Kuo:** Writing – review & editing, Writing – original draft, Formal analysis, Data curation, Conceptualization. **Vivia V. McCutcheon:** Writing – review & editing, Methodology. **Kathleen K. Bucholz:** Writing – review & editing, Methodology, Funding acquisition. **Danielle M. Dick:** Writing – review & editing, Funding acquisition. **Fazil Aliev:** Writing – review & editing, Data curation. **Jacquelyn L. Meyers:** Writing – review & editing, Funding acquisition. **Sarah J. Brislin:** Writing – review & editing. **Grace Chan:** Writing – review & editing. **Howard J. Edenberg:** Writing – review & editing, Funding acquisition. **Chella Kamarajan:** Writing – review & editing. **John Kramer:** Writing – review & editing. **Samuel Kuperman:** Writing – review & editing. **Dongbing Lai:** Writing – review & editing. **Martin H. Plawecki:** Writing – review & editing. **Carolyn E. Sartor:** Writing – review & editing. **Marc A. Schuckit:** Writing – review & editing. **Jessica E. Salvatore:** Writing – review & editing, Writing – original draft, Funding acquisition, Conceptualization.
