# Supplementary material for: The Impact of Polygenic Risk, Parental Separation, and Parental Relationship Discord on Heavy Episodic Drinking Across Adolescence and Young Adulthood in a High-Risk Sample
Source: JAACAP Open. 2025 Jun 9;3(4):1177–87. doi: 10.1016/j.jaacop.2025.06.001 (PMC12684652; doi:10.1016/j.jaacop.2025.06.001)
Supplement: Supplementary Material [file mmc1.pdf]

Supplementary Materials for
"The impact of polygenic risk, parental separation, and parental relationship discord on heavy episodic drinking across adolescence and young adulthood in a high-risk sample"

Correspondence to: sally.kuo@rutgers.edu

Perceptions of parental relationship discord. Participants reported on their perceptions of parental relationship discord using 5 questions. These items asked participants about the quality of their parents' marriage/relationship (rated on a 4-point scale, from *poor* to *excellent*) and how much conflict or tension there was in the household (rated on a 4-point scale, from *none* to *a lot*); and three yes/no questions: whether their parents usually seemed to enjoy each other; whether their parents often argued or fought in front of them; and whether either of their parents ever hit the other. Response options were 0 (*no*) and 1 (*yes*) for binary items. For other questions, the response was made using a 4-point ordinal scale. To create a composite measure, items were rescaled to a 0-1 range (0, 0.33, 0.66, 1) to standardize the weighting of ordinal and binary items within the prorated sum. Given the pattern of missing data (46.5% complete data, 22% missing one item, 0.2% missing two, 0.1% missing three, 22.3% missing four, and 8.9% missing all five), participants who responded to at least three of the five items were included in the composite score calculation by taking the prorated sum of the items (Thomas et al., 2023).

Thomas, N. S., Salvatore, J. E., Kuo, S. I., Aliev, F., McCutcheon, V. V., Meyers, J. M., ... & COGA Collaborators (2023). Genetic nurture effects for alcohol use disorder. *Molecular Psychiatry*, *28*(2), 759-766.

Genotyping. Participants' DNA samples were genotyped using the Illumina Human1M array (Illumina, San Diego, CA), the Illumina Human OmniExpress 12V1 array (Illumina), the Illumina 2.5M array (Illumina) or the Smokescreen genotyping array (Biorealm LLC, Walnut, CA; Baurley et al., 2016); a full description of data processing, quality control, and imputation is available in Lai et al. Data were imputed to 1000 Genome Phase 3, and single nucleotide polymorphisms (SNPs) with a genotyping rate < 0.95, that violated Hardy-Weinberg equilibrium ($p < 10^{-6}$), or had minor allele frequency (MAF) < 0.01 were excluded from analysis.

Lai, D., Wetherill, L., Bertelsen, S., Carey, C. E., Kamarajan, C., Kapoor, M., ... & Foroud, T. (2019). Genome-wide association studies of alcohol dependence, DSM-IV criterion count and individual criteria. *Genes, Brain and Behavior*, *18*(6), e12579.

Parental education. Parental education was measured using parent-reported data on the highest level of education completed, based on the response to the question "What is the highest grade in school you completed?" Responses were converted to the number of years typically required to complete that level of education, ranging from 0 to 17 years: primary or secondary school = actual year; technical school/ 1 year college = 13 years; 2 years college = 14 years; 3 years college = 15 years; 4 years college = 16 years; any graduate degree = 17 years). If data were available for both parents, the highest level was used; if only one parent's data was available, that value was used.

Supplemental Table S1. Mean and standard deviation of frequency of past-year heavy episodic drinking by age in the EA and AA samples

	EA		AA	
Age	Mean	SD	Mean	SD
12	0.38	4.83	0.00	0.00
13	0.15	1.32	0.38	4.02
14	1.24	9.67	0.33	3.23
15	4.38	19.90	0.75	4.56
16	9.31	30.46	3.52	17.41
17	14.67	36.52	5.42	26.69
18	20.60	46.14	8.81	30.60
19	29.97	55.72	17.10	43.02
20	35.55	62.66	15.59	40.96
21	39.23	60.51	16.61	42.69
22	44.37	66.52	16.47	40.56
23	39.54	63.61	22.85	47.81
24	37.37	60.57	25.02	64.00
25	31.52	54.44	27.17	54.03
26	31.02	56.09	23.22	56.39
27	28.86	57.71	22.10	54.85
28	28.52	57.28	28.64	64.97
29	22.46	44.86	19.21	52.73
30	19.71	44.32	26.97	58.73
31	26.39	56.73	24.74	65.74
32	18.81	43.05	29.18	74.75

Notes: Frequency of heavy episodic drinking was measured in days in the past 12 months.

Supplemental Table S2. Zero-order correlations (EA sample lower triangle, AA sample upper triangle)

	1	2	3	4	5	6	7	8
1 Age at first assessment	-	**0.59**	0.04	-0.02	0.06	-0.04	0.03	**0.13**
2 Age at last assessment	**0.58**	-	**-0.09**	0.05	**0.24**	-0.02	-0.06	-0.01
3 Sex (male)	-0.04	**-0.11**	-	0.05	**-0.10**	0.00	**-0.11**	-0.02
4 Parental education	**-0.11**	0.01	0.04	-	0.02	**-0.11**	**-0.18**	-0.02
5 Alcohol initiation	**-0.08**	**0.16**	-0.03	**0.15**	-	-0.04	-0.07	-0.07
6 PGS_{PAU}	-0.01	**-0.08**	-0.03	**-0.16**	**-0.13**	-	0.03	-0.02
7 Parental separation	**0.11**	-0.00	0.01	**-0.23**	**-0.22**	**0.06**	-	**0.23**
8 Parental discord	**0.22**	**0.11**	**-0.09**	**-0.16**	**-0.17**	**0.09**	**0.31**	-

Abbreviations: EA = genetically inferred continental groups of European American; AA = genetically inferred continental groups of African American; Parental education = highest levels of educational attainment (years) of either parent; alcohol initiation = age at initiation of regular drinking; PGS_{PAU} = problematic alcohol use polygenic scores. Bold type indicates estimate $p < .05$.

Supplemental Table S3. Linear mixed models predicting frequency of heavy episodic drinking across time as a function of parental history of AUD, parental separation, and parental discord in the EA sample

		Parental Separation Model N = 1751 Observations = 8262		Parental Discord Model N = 1362 Observations = 6600	
Parameters		b	95% CI	b	95% CI
Age	I	**-0.491**	**[-0.678, -0.303]**	**-0.415**	**[-0.606, -0.225]**
	S	**0.300**	**[0.258, 0.342]**	**0.293**	**[0.250, 0.336]**
	Q	**-0.011**	**[-0.013, -0.009]**	**-0.011**	**[-0.013, -0.008]**
Sex (male)	I	**-0.238**	**[-0.410, -0.066]**	**-0.181**	**[-0.361, -0.002]**
	S	**0.174**	**[0.135, 0.214]**	**0.164**	**[0.121, 0.206]**
	Q	**-0.006**	**[-0.008, -0.004]**	**-0.005**	**[-0.007, -0.003]**
PH of AUD	I	0.069	[-0.129, 0.268]	0.069	[-0.131, 0.269]
	S	**0.077**	**[0.032, 0.122]**	**0.073**	**[0.026, 0.119]**
	Q	**-0.003**	**[-0.006, -0.001]**	**-0.003**	**[-0.006, -0.001]**
Separation/Discord	I	**0.366**	**[0.180, 0.552]**	**0.128**	**[0.032, 0.224]**
	S	**-0.066**	**[-0.108, -0.023]**	-0.008	[-0.028, 0.013]
	Q	0.001	[-0.001, 0.003]	0.000	[-0.001, 0.001]

Note. Abbreviations: AUD = alcohol use disorder. CI = confidence interval. PH of AUD = parental history of AUD. I = Intercept. S = Linear slope. Q = Quadratic slope. Separate models were run for parental separation and parental relationship discord.
Bold type indicates estimate $p < .05$.

Supplemental Table S4. Linear mixed models predicting frequency of heavy episodic drinking across time as a function of parental history of AUD, parental separation, and parental discord in the AA sample

		Parental Separation Model N = 886 Observations = 4094		Parental Discord Model N = 463 Observations = 2199	
Parameters		b	95% CI	b	95% CI
Age	I	**-0.353**	**[-0.586, -0.119]**	**-0.319**	**[-0.545, -0.093]**
	S	**0.196**	**[0.137, 0.255]**	**0.199**	**[0.140, 0.257]**
	Q	**-0.006**	**[-0.010, -0.003]**	**-0.007**	**[-0.010, -0.003]**
Sex (male)	I	-0.057	[-0.264, 0.150]	-0.155	[-0.400, 0.090]
	S	**0.067**	**[0.016, 0.118]**	**0.086**	**[0.022, 0.149]**
	Q	0.000	[-0.003, 0.002]	-0.001	[-0.004, 0.003]
PH of AUD	I	0.066	[-0.149, 0.281]	0.086	[-0.162, 0.334]
	S	-0.014	[-0.069, 0.041]	-0.015	[-0.080, 0.050]
	Q	0.001	[-0.002, 0.004]	0.001	[-0.003, 0.005]
Separation/Discord	I	0.013	[-0.199, 0.225]	-0.071	[-0.183, 0.041]
	S	0.011	[-0.043, 0.066]	**0.063**	**[0.034, 0.091]**
	Q	0.000	[-0.003, 0.003]	**-0.003**	**[-0.004, -0.001]**

Note. Abbreviations: AUD = alcohol use disorder. CI = confidence interval. PH of AUD = parental history of AUD. I = Intercept. S = Linear slope. Q = Quadratic slope. Separate models were run for parental separation and parental relationship discord.
Bold type indicates estimate $p < .05$.

Supplemental Figure 1

Predicted change in frequency of heavy episodic drinking from adolescence to young adulthood by parental separation and sex in the EA sample

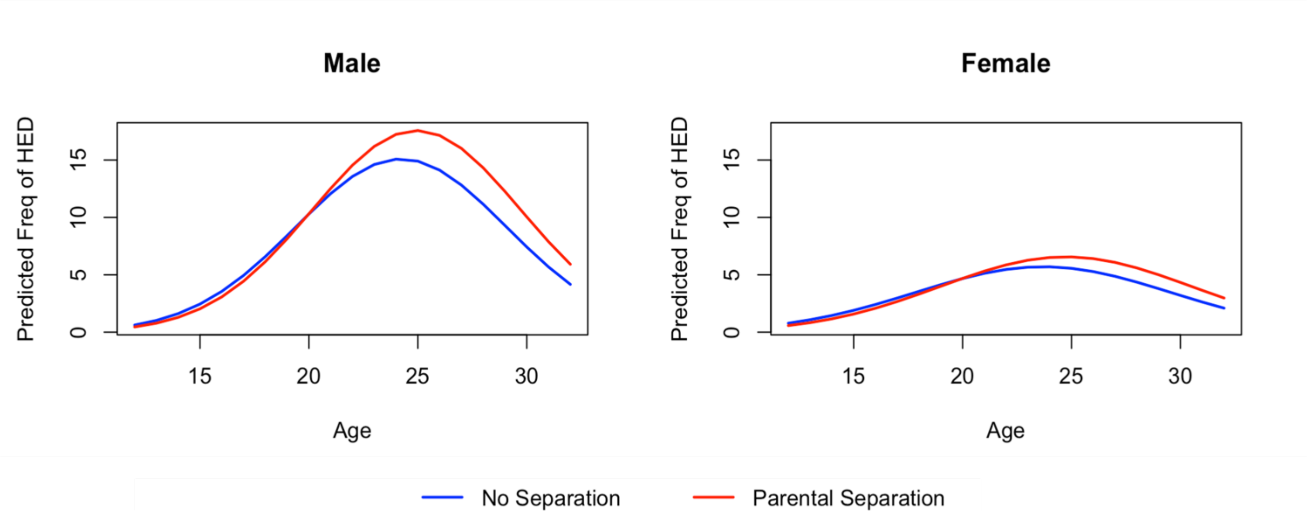

Notes: Abbreviation: HED = heavy episodic drinking. Figures depict the predicted model across sex and age. Residualized problematic alcohol use polygenic score was included on intercept and change over time in the predicted model.

Supplemental Figure 2

Predicted change in frequency of heavy episodic drinking from adolescence to young adulthood by parental relationship discord and sex in the AA sample

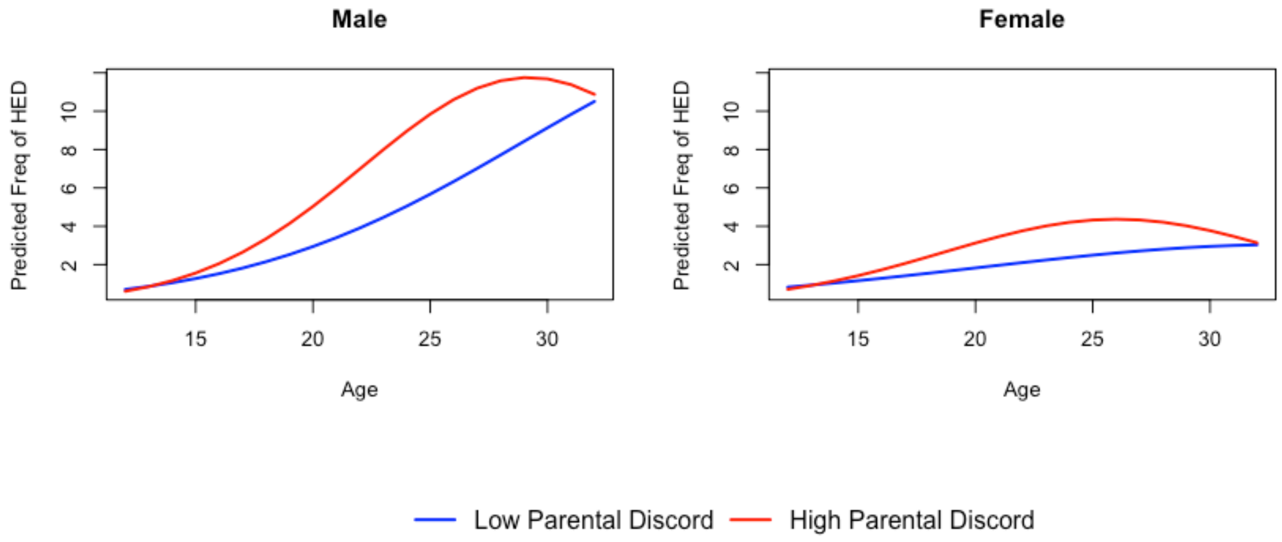

Notes: Abbreviation: HED = heavy episodic drinking. Figures depict the predicted model across sex and age. Residualized problematic alcohol use polygenic score was included on intercept and change over time in the predicted model. Parental relationship discord was fixed at -1*SD* and +1*SD*
